# Indirect Estimation of Heavy Metal Contamination in Rice Soil Using Spectral Techniques

**DOI:** 10.3390/plants13060831

**Published:** 2024-03-14

**Authors:** Liang Zhong, Shengjie Yang, Yicheng Rong, Jiawei Qian, Lei Zhou, Jianlong Li, Zhengguo Sun

**Affiliations:** 1 State Key Laboratory of Pharmaceutical Biotechnology, School of Life Sciences, Nanjing University, Nanjing 210023, China; zhongliang1007@163.com (L.Z.);; 2 Department of Ecology, School of Life Sciences, Nanjing University, Nanjing 210023, China; 3 Livestock Development and Promotion Center, Linyi 276037, China; 4 College of Agro-Grassland Science, Nanjing Agricultural University, Nanjing 210095, China

**Keywords:** rice, soil-crop system, heavy metal contamination, spectral technique, genetic algorithm, indirect estimation

## Abstract

The rapid growth of industrialization and urbanization in China has led to an increase in soil heavy metal pollution, which poses a serious threat to ecosystem safety and human health. The advancement of spectral technology offers a way to rapidly and non-destructively monitor soil heavy metal content. In order to explore the potential of rice leaf spectra to indirectly estimate soil heavy metal content. We collected farmland soil samples and measured rice leaf spectra in Xushe Town, Yixing City, Jiangsu Province, China. In the laboratory, the heavy metals Cd and As were determined. In order to establish an estimation model between the pre-processed spectra and the soil heavy metals Cd and As content, a genetic algorithm (GA) was used to optimise the partial least squares regression (PLSR). The model’s accuracy was evaluated and the best estimation model was obtained. The results showed that spectral pre-processing techniques can extract hidden information from the spectra. The first-order derivative of absorbance was more effective in extracting spectral sensitive information from rice leaf spectra. The GA-PLSR model selects only about 10% of the bands and has better accuracy in spectral modeling than the PLSR model. The spectral reflectance of rice leaves has the capacity to estimate Cd content in the soil (relative percent difference [RPD] = 2.09) and a good capacity to estimate As content in the soil (RPD = 2.97). Therefore, the content of the heavy metals Cd and As in the soil can be estimated indirectly from the spectral data of rice leaves. This study provides a reference for future remote sensing monitoring of soil heavy metal pollution in farmland that is quantitative, dynamic, and non-destructive over a large area.

## 1. Introduction

Soil serves both as the basis for the growth of crops and as a vital natural resource for the sustenance and production of human beings [1]. However, soil environmental pollution has increased significantly in China due to rapid industrialization and urbanization [2]. Among various pollutants, soil heavy metal contamination stands out due to its slow migration, high toxicity, and irreversible nature [3]. Over time, heavy metals accumulate in the food chain and pose severe health risks when ingested and accumulated by humans [4,5]. According to the 2014 Chinese Soil Pollution Status Report, the overall pollution excess rate of soil in China is 16.1% [6]. This alarming statistic has profound implications for China’s food security as heavy metal pollution in soil leads to an annual loss of approximately 12 million tons of grain crops [7]. As a result, both the government and scholars have shown widespread concern regarding soil heavy metal pollution [8,9]. Quantitative monitoring of heavy metal content plays an important role in understanding the extent and sources of heavy metal pollution in a region [10]. It also offers a theoretical foundation for the remediation and management of such pollution [11,12]. While traditional chemical analysis methods are highly accurate in detecting soil heavy metal content, they are time-consuming, labor-intensive, and costly. Consequently, they fail to support the demands of real-time and large-scale monitoring for efficient heavy metal content assessment [13]. Fortunately, the advancement of remote sensing technology has paved the way for nondestructive and rapid soil heavy metal monitoring using spectral remote sensing [14,15]. This technique, characterized by its high-resolution and ability to capture details of the object’s spectral information, holds great potential in enabling fast and efficient monitoring of soil heavy metal content [16].

Spectral remote sensing applications for monitoring soil heavy metal contamination include two main approaches: direct and indirect. Direct monitoring focuses on the mechanism where soil heavy metal are adsorbed by soil organic matter, iron-manganese oxides and clay minerals [17]. These components affect soil spectral morphology and reflectance, leading to specific soil spectral absorption features. At present, more research has been conducted on direct monitoring [18,19,20,21,22]. The direct monitoring method is not without its limitations, although it results in high model accuracy and stable models. Firstly, it is a time-consuming and cumbersome process that requires field soil sampling and laboratory processing to obtain soil spectral data. In addition, due to soil drying, grinding, and sieving, the spectral features of heavy metals extracted from laboratory soil spectra often differ from those obtained from remote sensing images. This difference complicates the direct application of these models to large-scale soil pollution monitoring using aerospace images [23]. Therefore, a more convenient and practical approach for widespread application is to use spectral data from plant leaves or canopies for indirect estimation of soil heavy metal contamination [24]. The approach is based on the principle that heavy metals move from the soil to the plants, accumulating there [25]. Under the stress of heavy metals, the protein and chlorophyll content of the plants are affected, which leads to a discernible difference in the reflectance spectra [26,27]. Related research has made significant progress. For example, Shi et al. [28] developed a multivariate spectral vegetation index based on rice canopy spectra for the estimation of arsenic (As) in farmland soil. Zhong et al. [29] estimated leaf Cu content using leaf hyperspectra and then inverted the Cu content of other parts of wheat and soil using bioconcentration factors. Wang et al. [30] observed that wheat canopy Cu increased with increasing soil Cu concentration, accompanied by distinct variations in spectral reflectance, providing a foundation for indirect estimation of soil Cu conten. However, the feasibility and accuracy of indirect estimation of soil heavy metals from rice leaves are not yet clear.

In this study, we will explore the potential of rice leaf spectra for estimating soil heavy metals. Firstly, we collected soil samples from farmland and also measured the spectral data of rice leaves in Xushe Town, Yixing City, Jiangsu Province, China. Then, we processed the leaf spectra with various spectral transformations and screened the spectral feature bands using a genetic algorithm (GA). Next, we used partial least squares regression (PLSR) to model soil Cadmium (Cd) and As content for for the spectra after different pre-processing. Finally, we evaluated the accuracy of the models and obtained the best estimation models.

## 2. Results

### 2.1. Statistics of Soil Samples

**Figure 1:**
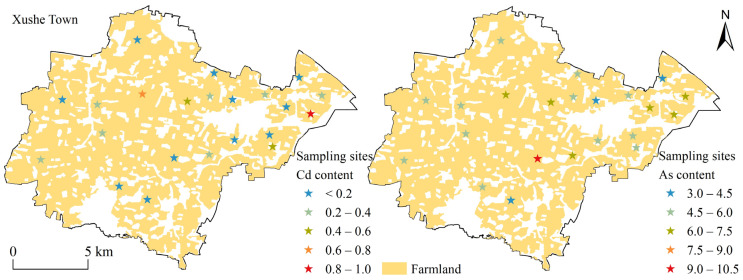
Distribution of Cd and As content at sampling sites in the study area.

Figure 1 shows the content of the soil Cd and As of the sampling sites in the study area. The average value of soil pH at the sampling site was 5.86. The Cd content was between 0.13 to 0.97 mg kg^−1^ with a average value of 0.29 mg kg^−1^, and the high values were located in the eastern and central parts of the study area. The As content was between 3.23 to 9.32 mg kg^−1^ with a average value of 5.64 mg kg^−1^, and the high values were mainly located in the central part of the study area. The correlation coefficient between Cd and As content at the sampling sites was 0.33.

### 2.2. Characterization of Spectral Curves of Rice Leaves

**Figure 2:**
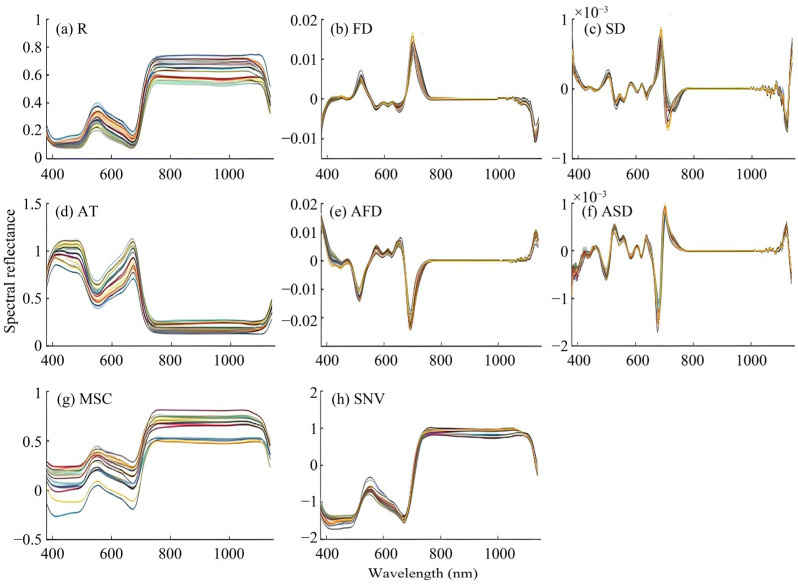
Characteristics of the spectral curves of rice leaves with different pre-processing.

Figure 2 shows the spectra of rice leaves after different pre-processing. From the raw spectra (R) (Figure 2a), it can be seen that the spectral curve of rice leaves is significantly different in the 760–1120 nm band. It has a green light reflection peak at 550 nm, blue-violet light absorption valleys at 450 nm, and red light absorption valleys at 670 nm. This is due to the fact that chlorophyll absorbs weakly in the green band of light and strongly in the blue-violet and red bands of light. There is a high reflectance in the 760 nm to 1120 nm near-infrared band, which may be caused by multiple reflections within the leaf structure.

Compared with R, the shape of the first-order derivative (FD) spectrum curve (Figure 2b) changed significantly, with a valley of absorption at 1129 nm and peaks of reflectance at 516 nm and 705 nm. The shape of the second-order derivative (SD) spectrum curve (Figure 2c) also changed significantly, with valleys of absorption at 712 nm and 1119 nm and peaks of reflectance at 503 nm and 686 nm. The absorbance transformation (AT) spectrum (Figure 2d) has high reflectance in the 400–510 nm band, low reflectance in the 730–1100 nm band, a valley of absorption at 553 nm, and a peaks of reflectance at 673 nm. The first-order derivative of absorbance (AFD) spectrum (Figure 2e) has valleys of absorption at 516 nm and 693 nm, and a reflection peak at 572 nm. The second-order derivative of absorbance (ASD) spectrum (Figure 2f) has valleys of absorption at 503 nm and 679 nm, and peaks of reflectance at 446 nm, 526 nm, 560 nm, 709 nm, and 1123 nm. The trend of the multiplicative scatter correction (MSC) spectrum curve (Figure 2g) is the same as that of the original spectrum, but the difference in the spectral curve of the 380–700 nm band is enlarged. The trend of the standard normal variate (SNV) spectrum curve (Figure 2h) is similar to that of the original spectrum, but the spectra are denser, indicating the ability of the SNV transform to reduce background noise.

### 2.3. Spectral Feature Bands Selected by GA

**Figure 3:**
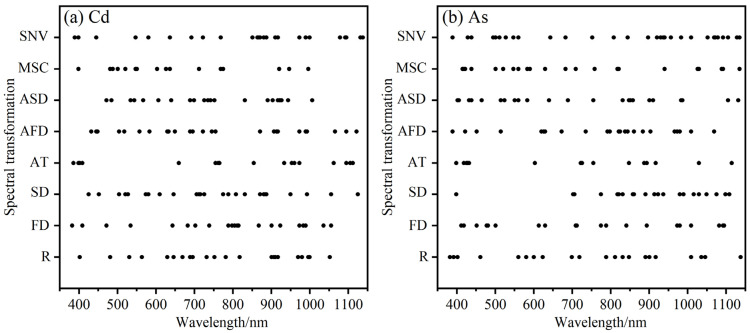
The feature bands of rice leaves spectral screened by GA.

Rice leaf spectral feature bands were screened using GA as shown in Figure 3. Under different spectral pre-processing, GA selected 17–25 feature bands of soil Cd and 15–30 feature bands of soil As among 230 full bands. The soil Cd feature bands are mostly concentrated in 400–410 nm, 520–580 nm, 630–650 nm, 690–770 nm, 870–925 nm, and 970–1000 nm; the soil Cd feature bands are mostly concentrated in 400–440 nm, 815–860 nm, 890–920 nm, 970–990 nm, 1005–1030 nm, and 1065–1135 nm.

### 2.4. Comparison of GA-PLSR and PLSR Modeling Results

Using the GA-PLSR and PLSR models, the modeling and analysis of rice leaf spectra under different pre-processing were carried out, and the cross-validation results are shown in Table 1. Compared with the PLSR model established directly using the full band, the PLSR model established by first screening the bands with GA has the same or reduced number of PCs, indicating that GA can select rice leaf spectral bands that are more meaningful to the PLSR model. Compared with the PLSR model, the *R*^2^_cv_ value of estimating soil Cd content under different pre-processing spectra using the GA-PLSR model increased by 6.25~33.96%, and the RMSE_cv_ value decreased by 0.00~50.00%. The *R*^2^_cv_ value of estimating soil As content increased by 14.29~53.19%, and the RMSE_cv_ value decreased by 3.33~69.64%. The results indicate that using GA for spectral wavelength selection before establishing a model for estimating heavy metal content in rice leaf spectra can improve model accuracy and stability.

### 2.5. Best Estimate Model

A cross-validation and an external validation were carried out on the GA-PLSR model to estimate the heavy metal content of the soil, and the results are presented in Table 2. The results of the soil Cd content estimation model show that, compared with R, the accuracy of each indicator was improved to different degrees in the cross-validation and external validation of the 7 transformed spectra. This indicates that the accuracy and stability of the Cd content estimation model have been improved after different spectral transformations. Among them, the RPD values for R, FD, SD, ASD, and MSC spectral preprocessing are all less than 1.50, suggesting poor accuracy in estimating soil Cd content. The RPD values of AT and SNV spectral preprocessing ranges between 1.50 and 2.00, indicating the possibility to discriminate between soil with high and low Cd content. The AFD spectral preprocessing has the highest model accuracy, with *R*^2^_cv_, RMSE_cv_, *R*^2^_ev_, RMSE_ev_, and RPD of 0.71, 0.07 mg kg^−1^, 0.77, 0.06 mg kg^−1^, and 2.09, respectively, indicating the ability to approximate soil Cd content estimation.

The results of the model for estimating soil As content show that the cross-validation and external validation of the seven transformed spectra improve the accuracy of each index to varying degrees compared to R. This indicates that the different spectral transformations have improved the accuracy and stability of the As estimation models. Among them, the R, FD, SD, AT, ASD, and MSC spectral preprocessing have an RPD values between 1.50 and 2.00, indicating the possibility of distinguishing between soil with high and low As content. The RPD values of SNV spectral preprocessing is 2.06, indicating the ability to approximate soil As content estimation. The AFD spectral preprocessing has the highest level of model precision, with *R*^2^_cv_, RMSE_cv_, *R*^2^_ev_, RMSE_ev_, and RPD of 0.89, 0.34 mg kg^−1^, 0.89, 0.30 mg kg^−1^, and 2.97, respectively, indicating good ability to estimate soil As content.

## 3. Discussion

### 3.1. Effect of Spectral Pre-Processing and Feature Selection for Modeling Performance

Modelling results from different spectral preprocessing techniques show that most preprocessed spectra show varying degrees of accuracy improvement over the original spectra. This improvement can be attributed to the fact that external disturbances can introduce noise when collecting spectral data, making it difficult to accurately represent the spectral characteristics of objects [31]. Spectral pre-processing approaches efficiently reduce spectral noise and improve the information about the spectral features [32,33]. For indirect soil heavy metal content estimation using rice leaf spectra, AFD is the optimal spectral transformation method for both Cd and As estimation. This is because of the FD spectral transform, which can effectively extract and enhance the hidden information in the spectrum [34]. Yao et al. [35] found that derivative transformations were able to highlight the spectral features more compared with MSC, SNV and Continuum removal. Meanwhile, the absorbance transformation can further improve the inversion accuracy of As content, which is consistent with our ability to obtain a good estimation of soil As content using AFD spectra.

In this study, the number of feature bands selected by GA in the optimal inversion models for soil Cd and As contents were 25 and 23, respectively, only about 10% of the bands were used. Moreover, higher accuracy was achieved by utilizing GA for selecting spectral characteristic bands compared to PLSR modelling. The reason for this approach is that spectral data have properties of redundancy and collinearity, and direct modelling with PLSR is susceptible to being disturbed by significant amounts of redundancy information [36]. GA improves model quality and stability by successfully filtering feature bands from the full spectrum [37]. The results of Sun and Zhang [38], Sun et al. [39], and Zhong et al. [17], who also showed that GA-PLSR outperforms PLSR in estimating heavy metal concentration using soil spectral data, are consistent with this methodology. It indicates that the method applied to the spectral modelling of heavy metal content in agricultural soils. In addition, Zhang et al. [40] in the estimation of soil heavy metal Cd, the accuracy of *R*^2^ was 0.88 by PLSR modelling using soil spectral features associated with organic matter extracted using GA. Wei et al. [41] in the estimation of soil heavy metal As, the accuracy of *R*^2^ was 0.82 and 0.70 for Honghu City and Daye City in Hubei Province, China, after selecting the feature bands by using the stable competitive adaptive reweighting sampling algorithm coupled the successive projections algorithm followed by PLSR modelling. Bian et al. [42] in the estimation of a variety of soil heavy metals, PLSR and extreme learning machine models obtained the best accuracy for Cd (*R*^2^ of 0.89) and As (*R*^2^ of 0.86), respectively. These studies generally obtained good model accuracies, but they mainly used laboratory soil spectra for direct monitoring of soil heavy metal content. Our study used rice leaf spectra to indirectly estimate soil heavy metal Cd and As contents, which is of significant value in the future development of field soil heavy metal hyperspectral instrumentation and in the exploration of aerospace hyperspectral remote sensing for monitoring soil heavy metal contamination on a large scale.

### 3.2. Application and Perspectives of Spectral Techniques in Heavy Metal Inversion in Soil-Rice System

Spectral data are characterized by high spectral resolution, which can obtain fine spectral information about the object. Using spectral analysis technology, it can highlight the subtle differences between spectra, which is conducive to extracting spectral features and finally constructing models to invert the information of the object [23]. In the soil-crop system, heavy metals migrate from the soil and accumulate in the crop. When the concentration of heavy metals in the soil increases, the content of heavy metals in different parts of rice usually increases as well, resulting in a stress effect [43]. As the stress of heavy metals in rice increases, some of the cellular structures are damaged, resulting in a decrease in chlorophyll content, which is reflected in differences in leaf spectra [44,45]. Therefore, we successfully inverted the content of heavy metals (Cd and As) in soil by spectral transformation, characteristic analysis, and modeling of rice leaf spectra. This study can help to develop soil heavy metal monitoring instruments in the future directly through rice leaf spectra, and thereby improve the detection efficiency of soil heavy metals. Meanwhile, in the future, attempts can be made to expand this indirect monitoring approach to aerospace spectral remote sensing, further combining space-air-ground spectral remote sensing data. This is conducive to making full use of the characteristics and laws of ground-based spectral indirect monitoring, while giving full play to the characteristics of aerospace spectral remote sensing in terms of dynamics and wide range. This will be helpful to form a multi-scale monitoring and validation system for soil-crop heavy metal pollution [17].

### 3.3. Limitations and Future Work

In this study, we used rice leaf spectra to indirectly estimate the content of soil heavy metals Cd and As, but there are still some limitations that we would like to explore and improve in future work, such as: (1) We studied and tested our method based on spectral data at only one sampling time, and the stability and applicability of the method need to be further validated. In the future, the indirect estimation method of soil heavy metal content in this study can be further explored for application at different time and spatial scales. (2) We only selected two typical pollutant elements in the study area for our study, and the potential of other heavy metal elements in spectral monitoring needs to be further explored in subsequent studies. (3) The mechanisms of uptake, transport and accumulation of heavy metal elements in the soil-crop system can be further explored in the future, which will provide more basis for the indirect monitoring of soil heavy metal content using crop spectra.

## 4. Materials and Methods

### 4.1. Study Area

**Figure 4:**
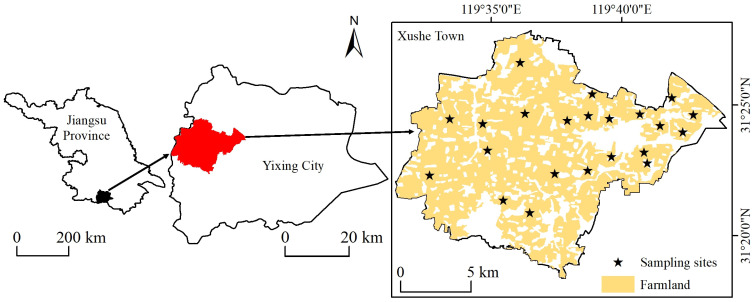
Location of the study area and distribution of sampling sites.

The study area is in the town of Xushe (31°18′–31°27′ N, 119°31′–119°44′ E), west of Yixing City, Jiangsu Province (Figure 4), and the total area is about 1.8 × 10^4^ hm^2^. The region features a subtropical monsoon climate with well-defined seasons, ample rainfall, and an average annual temperature of 16.0 °C, accompanied by precipitation of 1434.0 mm. The topography of the region is characterized by higher elevation in the western areas and lower elevation in the eastern areas, consisting mainly of plains and hills. With a cultivated area of 1.2 × 10^4^ hm^2^, Xushe Town is the largest agricultural town in Yixing City, primarily used for cultivating rice and wheat. Paddy, fluvo-aquic and yellow-brown soils are the three main soil types.

### 4.2. Data Collection and Processing

#### 4.2.1. Soil Sampling and Data Determination

We collected 22 surface (0–20 cm) soil samples in September 2019 in the study area using a five-point mixing method (Figure 3). At the time of sampling, we determined and recorded the location of each sampling point using a hand-held GPS device. The soil samples were then returned to the laboratory in sealed bags. In the laboratory, all soil samples were dried, ground and sieved (0.15 mm pore size) and divided equally into two parts. A part of the soil samples was used for the measurement of the pH by the potentiometric method (NY/T 1377-2007) [46]. The other part was weighed at 0.2 g of soil sample, put into the bottom of the PTFE digestion tank, added 5 mL of nitric acid, 2 mL of hydrogen peroxide, and 2 mL of hydrofluoric acid, and microwaved digested for 15 min. After the digestion solution was clarified, it was fixed to 50 mL and filtered. Finally, the Cd and As contents were determined by inductively coupled plasma mass spectrometry (ICP-MS) [47].

During the soil sampling process, we collected spectral data of rice leaves using a portable field spectrometer (UniSpec, PP systems, Haverhill, MA, USA) between 11:00 a.m. and 2:00 p.m. Beijing time. The spectrometer had a spectral range of 301–1145 nm and a spectral resolution of 3.3 nm. At each sampling site, five rice plants were randomly selected, and three fully expanded leaves from each rice were selected for spectral measurement under sunny and light wind conditions. White calibration was carried out before each spectral measurement and five measurements were repeated. For each sampling site, 75 spectral data points were collected from rice leaves and averaged to obtain the spectral data.

#### 4.2.2. Spectral Pre-Processing

Firstly, the spectral data of the rice leaf are stripped of the noisy edge bands below 380 nm. At each sampling site, the bands between 380 and 1145 nm are selected for spectral data processing and analysis. Next, the leaf spectral data of rice is processed using Savitzky-Golay smoothing. The smoothed spectral data is referred to as raw spectrum R. Finally, based on the R spectra, the spectral pre-processing was carried out by applying mathematical transform methods such as AT, SNV, MSC, FD, AFD, SD, and ASD [48].

### 4.3. Research Methods

#### 4.3.1. Genetic Algorithm

The GA is an evolutionary algorithm used for solving optimization problems. It simulates the mechanisms of genetics and natural selection, assessing individuals with superior fitness, selecting, crossing, and mutating them using genetic operators to generate individuals in the new generation of the population. In order to find the optimal solution, the iterative process is repeated until the convergence criteria have been met [49]. Simultaneously, GA can avoid the overfitting problem of general iterative methods, which may fall into the local minimum.

Prior to modeling, feature bands are selected using GA in order to reduce redundancies and optimize model performance [50]. In feature selection using GA, each band is treated as a gene, and a specific number of bands are designated as chromosomes. Next, a subset of the samples is taken to form the initial population. Then, crossing and mutation are used to simulate the genetic and evolutionary processes of random populations in nature, while the fitness function is used to assess the model’s performance in predicting outcomes. After conducting tests, the GA parameters for population size, crossover probability, mutation probability, and genetic generation were set to 40, 0.5, 0.01, and 100, respectively. We repeated the process 10 times to minimize the influence of randomness. We used the root mean squared error of cross-validation (RMSE_cv_) for PLSR as the fitness criterion. As the individual’s fitness increases, the RMSE_cv_ decreases.

#### 4.3.2. Partial Least Squares Regression

The PLSR is one of the most commonly used methods for processing spectral data to estimate soil heavy metal content [51]. In this method, the independent variable and the dependent variable are projected onto a new coordinate system. The principal component, which has the strongest explanatory power, is extracted and used to construct a new linear model. This helps reduce collinearity and noise effects and makes the model more robust [52]. During the process of PLSR modeling, cross-validation is utilized to identify the number of most efficient principal components.

The spectra after different pre-processing were used to select the characteristic bands using GA and estimate the heavy metal content using PLSR. The 22 data samples are divided into two parts, with one sample selected from every 4 samples for validation. In all, 17 samples were used for modeling, and 5 samples were used for validating the accuracy of the model. The GA feature band selection and the PLSR modelling were done in R-Studio 3.5.3 (https://posit.co/products/open-source/rstudio/ (accessed on 6 November 2019)).

#### 4.3.3. Model Assessment

This study used the coefficient of determination (*R*^2^_cv_) and the RMSE_cv_ for model cross-validation. The *R*^2^_ev_, RMSE_ev_, and relative percent difference (RPD) were chosen for model external validation. The closer *R*^2^_cv_ and *R*^2^_ev_ are to 1, the lower RMSE_cv_ and RMSE_ev_ are, and a higher RPD indicates a better model fit and accuracy. The five-layer interpretation method proposed by Williams et al. [53] was adopted for the evaluation criteria of RPD. If the RPD exceeds 3.00, the model has excellent ability to estimate. If the RPD ranges from 2.50 to 3.00, the model is considered to have good predictive performance. If the RPD ranges from 2.00 to 2.50, the model can be used for an approximate quantitative estimate. If the RPD ranges from 1.50 and 2.00, the model has the ability to discriminate between high and low values. If the RPD is less than 1.50, the model has a poor ability to estimate.

## 5. Conclusions

By combining spectral preprocessing, feature selection and modelling methods, this study fully explored the potential of rice leaf spectra for indirect estimation of soil heavy metals Cd and As, and the following conclusions were drawn:

(1) Spectral preprocessing technology enhances the modeling accuracy by revealing hidden information in the spectrum, leading to varying degrees of improvement compared to the original spectrum. When modeling rice leaf spectra, the most effective estimation models for soil Cd and As content are obtained through AFD spectral preprocessing. These results highlight the advantages of mathematical transformations, such as derivative transformation and absorbance, in extracting spectral sensitive information.
(2) The GA-PLSR model demonstrates superior performance compared to the PLSR model in modeling of rice leaf spectra. Specifically, compared to the PLSR model, GA-PLSR used only approximately 10% of the bands and enhanced the *R*^2^_cv_ values for estimating soil Cd and As content by 0.00% to 50.00% and 3.33% to 69.64%, respectively, for the different preprocessed spectra. These findings illustrate that incorporating a GA for spectral band selection before establishing a model for estimating soil heavy metal content can significantly enhance the accuracy and efficiency of the model.
(3) In the modeling of soil Cd content using rice leaf spectra, the best estimation model is the combination of AFD and GA-PLSR, with *R*^2^_ev_, RMSE_ev_, and RPD values of 0.77, 0.06 mg kg^−1^, and 2.09, respectively, which has the ability to approximate estimation. The best estimation model for soil As content is also the combination of AFD and GA-PLSR, with *R*^2^_ev_, RMSE_ev_, and RPD values of 0.89, 0.30 mg kg^−1^, and 2.97, respectively, which has good estimation ability.

## Figures and Tables

**Table 1:** Comparison of the accuracy of GA-PLSR and PLSR models for estimating soil Cd and As content in rice leaves.

Heavy Metal	Pre-Processing	Number of Bands	GA-PLSR			PLSR		
			PC	*R* ^2^_cv_	RMSE_cv_/(mg kg^−1^)	PC	*R* ^2^_cv_	RMSE_cv_/(mg kg^−1^)
Cd	R	22	2	0.34	0.16	2	0.32	0.18
	FD	21	3	0.46	0.15	3	0.39	0.15
	SD	25	1	0.42	0.15	2	0.35	0.17
	AT	17	2	0.53	0.15	2	0.45	0.15
	AFD	25	5	0.71	0.07	8	0.53	0.14
	ASD	22	2	0.47	0.13	4	0.38	0.17
	MSC	16	1	0.47	0.15	3	0.40	0.16
	SNV	26	2	0.52	0.15	2	0.45	0.15
As	R	21	2	0.50	1.18	3	0.41	1.27
	FD	20	1	0.52	1.15	2	0.42	1.22
	SD	21	2	0.55	1.16	3	0.44	1.25
	AT	15	2	0.56	1.16	2	0.49	1.20
	AFD	23	5	0.89	0.34	9	0.61	1.12
	ASD	23	2	0.72	0.82	6	0.47	1.44
	MSC	22	4	0.58	1.15	4	0.42	1.27
	SNV	30	2	0.70	0.93	6	0.51	1.17

**Table 2:** Accuracy of GA-PLSR models for estimating soil Cd and As content in rice leaves.

Heavy Metal	Pre-Processing	Cross-Validation		External Validation		
		*R* ^2^_cv_	RMSE_cv_/(mg kg^−1^)	*R* ^2^_ev_	RMSE_ev_/(mg kg^−1^)	RPD
Cd	R	0.34	0.16	0.41	0.11	1.30
	FD	0.46	0.15	0.52	0.10	1.44
	SD	0.42	0.15	0.47	0.10	1.37
	AT	0.53	0.15	0.59	0.09	1.56
	AFD	0.71	0.07	0.77	0.06	2.09
	ASD	0.47	0.13	0.53	0.09	1.46
	MSC	0.47	0.15	0.49	0.10	1.40
	SNV	0.52	0.15	0.62	0.08	1.62
As	R	0.50	1.18	0.57	0.65	1.52
	FD	0.52	1.15	0.68	0.56	1.77
	SD	0.55	1.16	0.66	0.58	1.71
	AT	0.56	1.16	0.64	0.60	1.66
	AFD	0.89	0.34	0.89	0.30	2.97
	ASD	0.72	0.82	0.71	0.53	1.86
	MSC	0.58	1.15	0.64	0.60	1.66
	SNV	0.70	0.93	0.76	0.48	2.06

## Data Availability

Data are contained within the article.
